# Strong attachment as an adaptation of flightless weevils on windy oceanic islands

**DOI:** 10.1098/rsif.2023.0447

**Published:** 2023-11-22

**Authors:** Lu-Yi Wang, Chung-Ping Lin, Stanislav N. Gorb, Hamed Rajabi

**Affiliations:** ^1^ School of Biosciences, Faculty of Science, The University of Melbourne, Melbourne, Australia; ^2^ Functional Morphology and Biomechanics, Institute of Zoology, Kiel University, Kiel, Germany; ^3^ Department of Life Science, National Taiwan Normal University, Taipei, Taiwan; ^4^ Mechanical Intelligence (MI) Research Group, South Bank Applied BioEngineering Research (SABER), School of Engineering, London South Bank University, London, UK; ^5^ Division of Mechanical Engineering and Design, School of Engineering, London South Bank University, London, UK

**Keywords:** adhesion, Curculionidae, cuticle, island biogeography, *Pachyrhynchus*, Taiwan

## Abstract

Enhanced attachment ability is common in plants on islands to avoid potential fatal passive dispersal. However, whether island insects also have increased attachment ability remains unclear. Here we measured the attachment of a flightless weevil, *Pachyrhynchus sarcitis kotoensis*, from tropical islands, and compared it with documented arthropods from the mainland. We examined the morphology and material gradient of its attachment devices to identify the specific adaptive modifications for attachment. We find that the weevil has much stronger attachment force and higher safety factor than previously studied arthropods, regardless of body size and substrate roughness. This probably results from the specific flexible bases of the adhesive setae on the third footpad of the legs. This softer material on the setal base has not been reported hitherto and we suggest that it acts as a flexible hinge to form intimate contact to substrate more effectively. By contrast, no morphological difference in tarsomeres and setae between the weevil and other beetles is observed. Our results show the remarkably strong attachment of an island insect and highlights the potential adaptive benefits of strong attachment in windy island environment. The unique soft bases of the adhesive hairs may inspire the development of strong biomimetic adhesives.

## 1. Introduction

High dispersibility is selected for cross-oceanic dispersal of island species; however, natural selection selects against it after landing [1]. Passive dispersal by wind is one of the main challenges for organisms on islands because individuals can be blown off to the sea and perish [1,2]. This is especially challenging for small organisms due to their lighter body weight. To prevent this, some island species take passive strategies by reducing flight frequency and/or turning into ground feeders, such as flightless insects, bats and birds [3–5]. By contrast, other species take active strategies by evolving more secured attachment. For example, herbaceous *Fitchia* plants grow into tree-like size and increase woodiness on the Polynesian islands [6]. Insular insects show a higher tendency to be flightless [7]; nevertheless, whether they also evolve strong attachment ability to prevent passive drift remains unknown.

Natural selection can shift functional traits of island organisms through extreme climatic events, such as tropical cyclones (i.e. typhoons or hurricanes). Tropical cyclones bring devastating winds at high speeds of 120 to greater than 250 km h^−1^ [8], which is a strong selective pressure on island species. This selective force favours individuals with greater attachment ability due to their higher likelihood to remain on islands during a cyclone. This is demonstrated by *Anolis* lizards on Caribbean islands—the populations that experience hurricanes more frequently have larger toepads [9], indicating stronger clinging ability [10]. Small animals, such as insects, may be more strongly subjected to this selective pressure because they are lighter and more easily displaced by wind.

Secured attachment is crucial for arboreal insects, because it not only provides stable movement above ground, but also avoids potential cost from falling to the ground. It is particularly important for *Pachyrhynchus* weevils (Coleoptera: Curculionidae: Entiminae: Pachyrhynchini), because these weevils are arboreal and flightless [11,12]. Dropping to the forest floor could expose them to the ground predators that are naive to their bright warning coloration or puddles of water that could be fatal for their tiny body size [13–15]. Most importantly, they mainly live on oceanic islands in the Old World tropics [11,16], where typhoons occur several times annually. How these flightless weevils living in the canopy survive severe windy events awaits investigation.

**Figure 1.**
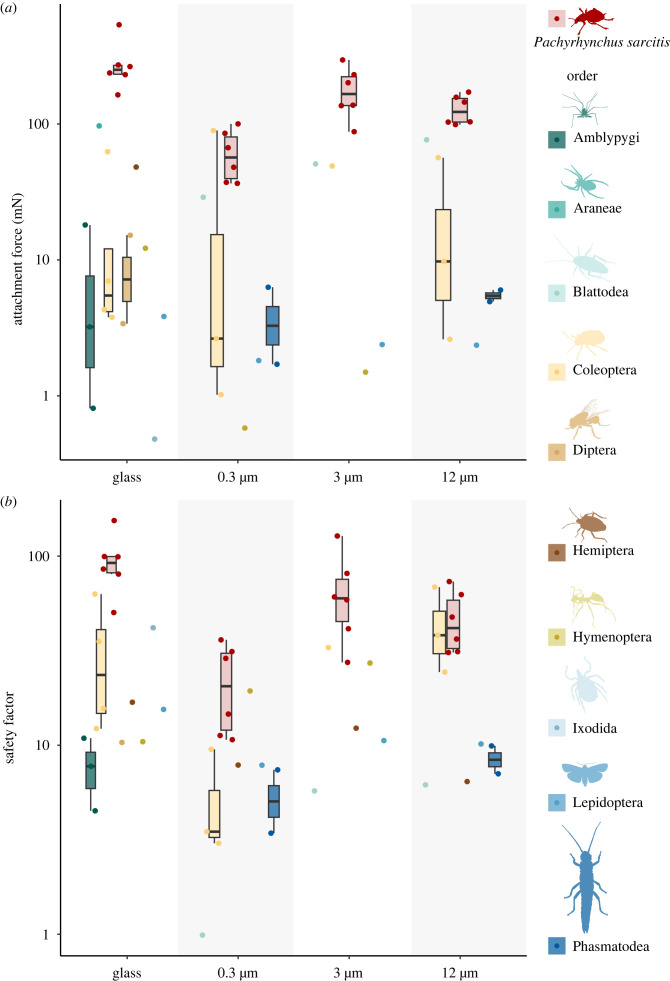
Comparison of attachment force (*a*) and safety factor (*b*) between *Pachyrhynchus sarcitis kotoensis* and the other arthropods (see electronic supplementary material, table S3) on various surface roughness. Both y-axes are log_10_ transformed. Each dot shows the data of different species obtained from the literature with different colours representing different orders while *P*. *sarcitis kotoensis* is in colour red. We plot box-and-whisker plots for orders that have more than three data points on each surface roughness. In each box-and-whisker plot, the middle line is the median, box limits are the upper and the lower quartiles, and whiskers are 1.5 times of the interquartile range.

Here we tested if the insular insects have stronger attachment than that of arthropods from the mainland using *Pachyrhynchus* weevils as study animals and investigated their attachment mechanism. Specifically, we measured the attachment force and safety factor (the load an individual can carry per unit of its weight) of *P. sarcitis kotoensis* on surfaces of different roughness. These measures of attachment ability were compared with previously documented values from other species of arthropods measured on similar surface roughness for reliable comparisons (figure 1). To test the contribution of tarsal claws to attachment, we repeated the same experiment on the weevils with surgically removed tarsal claws. To identify the adaptive modifications of the attachment devices, we examined the morphology and level of sclerotization of the weevil's tarsomeres (foot segments) and setae (hairs on the ventral side of the tarsomeres used for attachment purpose). We compared and discussed the morphological differences between the weevil and other beetle species. The exoskeleton of the elytra of teneral *Pachyrhynchus* weevils (freshly enclosed that come out of the pupal chamber) is not fully developed and very soft and easily deformable [14,17]. To test whether the attachment force changed at different development stages of the cuticle, we repeated the same experiment and microscopical examination on a teneral adult weevil for comparison.

## 2. Material and methods

### 2.1. Animals

All *Pachyrhynchus* species are endangered and protected in Taiwan and the Philippines (their major distribution region). Therefore, for this study we used only eight individuals from a single species, *Pachyrhynchus sarcitis kotoensis* Kôno 1930 to minimize our impact on its population (permit no. 1060241435, the Forestry Bureau, Council of Agriculture, Taiwan). The species was chosen because its host plants are easier to access. Eight adult individuals were collected from Orchid Island, Taiwan in November 2017 by hand. All collected individuals were mature adults (mean mass 276.8 mg), tested by pressing on their elytra to confirm they had a rigid exoskeleton [17]. Once a week, we supplied the weevils with the leaves of their known host plant, *Leea guineensis* (Leeaceae), or *L. sambucina* as a substitute. We reared one teneral weevil (130.8 mg) in the laboratory from the egg of the field-collected weevils following the rearing method in Huang *et al*. [18].

Due to the low weevil number, we focused on the interspecific variation rather than the intraspecific variation. We expected *Pachyrhynchus* weevils to show no sexual dimorphism in morphology and material gradients from our preliminary examinations. We also expected no sexual difference in attachment force because male *Pachyrhynchus* weevils hug the females (i.e. extending legs to females' abdomens) during copulations rather attaching on females’ backs like leaf beetles [19].

### 2.2. Measurement of attachment force and safety factor

We measured the attachment force of the weevils on a series of four varied surface roughness using a centrifugal experiment set-up (see [20] for details of set-up). The roughness was selected based on those commonly used in the literature to enable reliable comparisons with previous studies though these surfaces were smoother than plant surfaces [21]. Here, we used glass and Spurr resin replicas of glass polished with sandpapers with asperity size of 0.3, 3 and 12 µm (10 mm diameter discs; see [22] for surface fabrication and [21,23] for details of surface profile). From here on, we will refer to these four surfaces as glass (smooth), 0.3 µm, 3 µm and 12 µm as they were termed in many other studies. These surfaces had average roughness (absolute surface height averaged over the surface) of 0.02, 0.35, 1.65 and 4.04 µm, respectively [21,23] (for comparison, leaf surface of *Vicia faba* is approximately 20 µm, [21]). The experiments were performed in the sequence from the smoothest to the roughest surface. The body mass of each weevil was measured before its first trial (AG204 Delta Range, Mettler Toledo, Columbus, OH, USA). In each measurement trial, we placed a weevil on a still disc and accelerated the rotation from 50 to 3000 r.p.m. in 20 s. The rotation stopped automatically upon the detachment of the weevil. We recorded the weevil's distance to the centre of the disc and the maximum angular velocity of the disc upon the detachment for force calculation. We used the vector addition of the centrifugal force and the tangential force as the maximum force generated by the weevil in the trial (see Gorb *et al*. [20] for formulae used) and calculated the corresponding safety factor (attachment force in mN/weight in mN). We repeated five trials on each roughness for each weevil (*n* = 6) with at least a 5 min break in between.

We used median force and safety factor for comparison with other taxa. To ensure a reliable comparison between the weevil's attachment ability and that of other mainland arthropods, we systematically selected references using the following steps (electronic supplementary material, table S3; [19,21,23–37]). We first selected studies that measured shear force using the similar methods as we used in the current study. Next, we filtered the attachment force and safety factor that were measured on the same surface roughness ranges because roughness affects the attachment force measured [38].

Additionally, we tested the effect of tarsal claws on attachment and tested if the attachment force differed when the cuticle was at different stages of sclerotization. Specifically, we repeated the same measurements on the same individuals, but with the tarsal claws surgically removed and on one teneral adult weevil for comparison. Full details are provided in electronic supplementary material, information S1 and S2, respectively.

### 2.3. Morphology and material gradients of attachment devices

To examine the morphology and level of sclerotization of the weevil's attachment devices, we examined the weevil's tarsomeres and setae by scanning electronic microscopy (SEM) and confocal laser scanning microscopy (CLSM), respectively. Specifically, we examined the tarsomeres and the setae of the fore-, mid- and hind-legs from one weevil. The weevils were fully thawed from −17°C to room temperature, and the tarsi were removed for sample preparation. For the SEM, the tarsi were dehydrated in an ascending ethanol series—ethanol concentration of 10% and 30% for 10 min each, followed by 50%, 70% and 90% for 15 min each. After dehydration, we air dried the samples and glued the tarsi on the aluminium SEM stubs (radius: 12.6 mm) with carbon adhesive tabs and sputter coated them with a thin layer of gold-palladium (thickness: approx. 9 nm) using a Leica EM SCD500 high-vacuum sputter coater (Leica Microscopy GmbH, Germany). We used a Hitachi S 4800 scanning electron microscope (Hitachi Ltd, Tokyo, Japan) at an accelerating voltage of 3.0 kV for sample visualization.

Material properties affect the ability of adaptation to surfaces and resilin is an elastic protein that can increase adaptability of attachment devices [39]. To understand the material characteristics of the weevil's attachment devices, we used CLSM to visualize the distribution of resilin in weevil cuticle through cuticle autofluorescence as an indication of the distribution of material composition. For the CLSM samples, we cleaned the tarsi in an ultrasonic bath for 15 min and embedded them in glycerol. The samples were imaged using Zeiss LSM 700 (Carl Zeiss Microscopy, Jena, Germany) with 5X objective lens (Zeiss Plan-Apochromat; numerical aperture: 0.8) for the whole tarsi and with 10X objective lens (Zeiss Plan-Apochromat; numerical aperture: 0.8) for close-ups of tarsomeres. The CLSM was equipped with four laser beams with different wavelengths (405, 488, 555 and 639 nm) and four corresponding filters (BP420–480, LP490, LP560 and LP640 nm). With these laser beam and filter combinations, we applied colour blue to the autofluorescence with the wavelength 420–480 nm, indicating non-sclerotized regions; colour green to wavelength greater than 490 nm, representing regions with medium amount of resilin (less sclerotized); colour red to wavelengths greater than 560 nm and greater than 640 nm, revealing less resilin (highly sclerotized) regions. For detailed descriptions of the CLSM techniques and interpretations, see Michels & Gorb [40].

To compare the morphology and level of sclerotization at different adult stages (teneral versus mature), we examined the same parts of the attachment devices of a teneral weevil using the same methods.

### 2.4. Statistical analysis

To test if the *Pachyrhynchus* weevil had stronger attachment ability than other previously studied arthropods, we performed Mann–Whitney *U*-tests on the attachment force and safety factor of the two groups (weevils versus arthropods). To test the effect of tarsal claws on attachment, we used paired *t*-tests on the attachment force and safety factor of intact and clawless weevils. All tests were done for different surface roughness (glass, 0.3 µm, 3 µm, 12 µm), respectively.

Additionally, to test the effect of surface roughness on attachment, we performed Kruskal–Wallis tests on the attachment force and safety factor of the weevils. Subsequent Dunn's *post hoc* tests with Bonferroni corrections (‘dunnTest’ function in the R package ‘FSA’, [41]) were used to test for pairwise differences between surfaces. All statistical analyses were performed in R 3.6.3 [42].

## 3. Results

### 3.1. Attachment ability

The weevil showed significantly stronger attachment force and higher safety factor than previously measured arthropods (associated with the mainland) (electronic supplementary material, table S3) on all surface roughness (figure 1). The attachment force of the weevil was stronger by 14-fold on the glass surface (*U* = 0, *p* < 0.001), fourfold on the 0.3 µm surface (*U* = 5, *p* < 0.05), sevenfold on the 3 µm surface (*U* = 0, *p* < 0.01), and sixfold on the 12 µm surface (*U* = 0, *p* < 0.01) than that of the arthropods. When body weight was taken into account, the safety factor of the weevil was higher by fivefold on the glass surface (*U* = 1, *p* < 0.001), threefold on the 0.3 µm surface (*U* = 3, *p* < 0.01), fourfold on the 3 µm surface (*U* = 1, *p* < 0.01), and twofold on the 12 µm surface (*U* = 8, *p* < 0.05). The maximum attachment force and safety factor of the weevil were 12% and 7% higher than the maximum values recorded for other arthropods on the same surface roughness (attachment force on 0.3 µm, the weevil: 100.14 mN, the potato beetle, *Leptinotarsa decemlineata*: 89.41 mN, [33]; safety factor on the 12 µm, the weevil: 73.40, the leaf beetle, *Gastrophysa viridula*: 68.56, [37].

The weevils generated the strongest attachment force and the highest safety factor on the glass surface and the lowest on the 0.3 µm resin surface (electronic supplementary material, figure S1). The attachment force and safety factor on the glass surface were 4.6 and 4.3 times significantly higher than that on the 0.3 µm surface, respectively (force: Kruskal–Wallis test, *χ*^2^ = 16.67, d.f. = 3, *p* < 0.001; Z = 4.00, *p*_adj_ < 0.001; safety factor: *χ*^2^ = 14.10, d.f. = 3, *p* < 0.01; Z = 3.80, *p*_adj_ < 0.001), while no significant difference was detected between other surfaces (electronic supplementary material, table S4). The decrease in attachment force and safety factor from glass to 0.3 µm surfaces followed by an increase on 3 µm surface is coherent with the results on the stink bug (*Nezara viridula*) and stick insects [27,31]. The results were qualitatively the same for the clawless weevils (electronic supplementary material, information S1). No significant difference was detected between the intact and clawless weevils in attachment force and safety factor (electronic supplementary material, figure S1).

The attachment force of the teneral weevil was weaker than that of the mature ones, while the safety factor was higher due to lighter body weight (electronic supplementary material, information S2; figure S2).

### 3.2. Morphology and sclerotization level of tenent setae

We followed the terminology in Stork [43] for morphological description, where the plate is the tip of a seta and the shaft is the elongate part that supports the plate.

**Figure 2.**
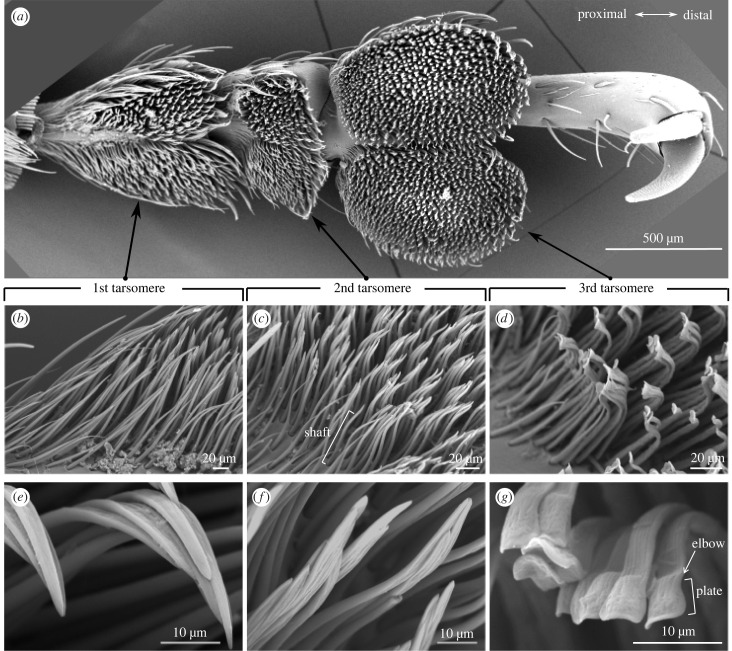
The morphology of the tarsomeres and the setae of *Pachyrhynchus sarcitis kotoensis*. (*a*) The tarsus of the hind-leg. (*b*)–(*d*) The geometry of the entire setae. (*e*)–(*g*) Close-ups of the setal tips.

From the SEM images, the setae on the first and second tarsomeres showed circular bases sloping distally (to the tarsal tip; figure 2*b,c*) and the terminal plates (spatulae) flattened into pointed tips from the joints. Setae also possessed elbows, the curved region connecting the plate and the shaft (figure 2*e,f*). By contrast, the setae on the third tarsomeres were shorter than those on the first two tarsomeres (figure 2*d*) and sharply bent proximally from the end of the shafts (around 1/20 of the shaft length). The shafts gradually flattened from the bases to the joints and regularly ribbed longitudinally along the dorsal side with occasional ridges on the ventral side. The regular ridges on the dorsal side reduce probability of neighbouring setae sticking together, which was previously reported for earwig spatulae [44], whereas the occasional ridges on the ventral side might be the result of some shrinkage of softer cuticle. The flat, spatula-like plates folded slightly toward the ventral sides and depressed centrally with the dorsal surfaces of the plates patchily foveolate. The third tarsomere had the largest pad area (0.73 mm^2^) followed by the first tarsomere (0.36 mm^2^) while the second tarsomere had the smallest pad area (0.18 mm^2^). The morphology was qualitatively similar for all legs.

**Figure 3.**
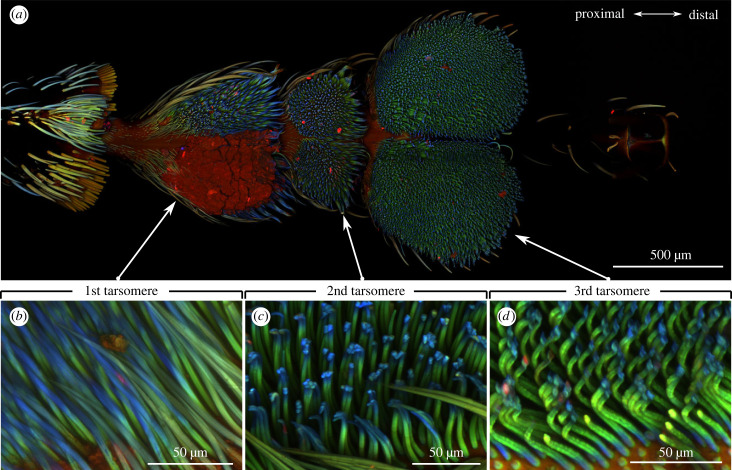
The level of sclerotization in the tenent setae of *Pachyrhynchus sarcitis kotoensis*. (*a*) The whole tarsus. Red patches on the first tarsomere are irremovable contamination. (*b*)–(*d*) The material gradient on the setae from the first to the third tarsomeres. Orange and yellow colours indicate highly sclerotized regions; green colour indicates less sclerotized regions; blue colour indicates elastic, resilin-dominated regions; black colour of the tarsomere indicates regions enriched by melanin.

From the CLSM images, the setae from the first and second tarsomeres showed green-fluorescing bases that gradually turned into short blue tips (figure 3*b,c*). This colour combination indicated relatively stiff sclerotized bases and soft non-sclerotized tips. By contrast, the setae on the third tarsomeres had short blue-fluorescing bases followed by green middles and short blue tips (figure 3*d*). The blue bases on the third tarsomeres indicated the flexibility in addition to the tips. The level of sclerotization was qualitatively similar for all legs.

## 4. Discussion

Here, we showed that the insular *P. sarcitis kotoensis* has very strong attachment force and high safety factor. Compared with the previously measured arthropods from the mainland, the weevil has around eight times stronger attachment force and four times higher safety factor. Such strong attachment ability is probably due to the unique soft setal bases that may function as an additional flexible hinge for enhancing adaptability to substrates. Combined, our results suggest that the impressive attachment ability of *Pachyrhynchus* weevils could be an important adaptive strategy to the windy environments of tropical islands or/and to avoiding small arthropod predators, such as ants, by strong adhesion to the substrate [45]. Both potential ecological functions of such strong adhesive system may be especially advantageous for flightless weevils. The soft bases of the hairs on the footpads, serving as additional joints for local adaptations of setae to the surface profile, may inspire new designs of stronger biomimetic adhesives (e.g. [46]).

The strong attachment of *P. sarcitis kotoensis* is probably enforced by the unique material distribution in addition to usual morphology of the tarsomeres and setae. This is because *P. sarcitis kotoensis* has the typical tarsomere shape and setal types of weevils, where the first two tarsomeres are inverted triangular with flat pointed setal tips, and the third tarsomeres are heart-shaped with spatula-like setal tips [43,47]. By contrast, for the material gradients, the first and second tarsomeres of the weevil have stiff setal bases and short soft setal tips, which is a general pattern in beetle's tarsomeres, e.g. Colorado potato beetles, seven-spot ladybirds and green dock beetles [39,48,49]. At the base of every seta in Coleoptera, there is a microjoint (socket) that provides flexibility and increases the adaptability to the substrate [46]. In addition to microjoints, a soft tip has been suggested to facilitate adaptability of the seta to substrate by serving as a flexible hinge that helps in forming intimate contact, regardless of the initial angle of contact [39,49]. On the other hand, the stiffer middle region has been suggested to prevent buckling of the slender setae and their clustering by enhancing their flexural stiffness [39,50]. Aside from the stiff middle regions and soft tips, the setae on the third tarsomeres of the weevil have additional unique soft bases, which have never been reported in other animals hitherto. Here we suggest that the soft setal base functions as a flexible hinge that could potentially increase the attachment additionally to the sockets and soft setal tips. It could enhance the effect of the hinge at the tip of the setae by enabling a higher deformability and the rotation of the whole seta body around the base. This could be especially important in contacts with uneven substrates, in which a higher deformability would provide better adaptability. Though this character is only found in the third tarsomeres, it still probably has an important contribution to the individual's attachment, as the third tarsomere is one and three times larger than the first and second tarsomeres, respectively.

The strong attachment force of the weevils may not only prevent the weevils from being blown away during storms but also enhance cross-island dispersal. In their natural habitats, the weevils experience different external forces that may weaken attachment, such as attacks from predators [14] or severe winds. Around 27 typhoons are formed annually in west North Pacific, which is the main distribution region of the *Pachyrhynchus* weevils, and each typhoon can last from 1 to greater than 10 days [51]. The common wind speed of typhoon generates a wind pressure of approximately 190 mN cm^−2^ [51]. Assuming the lateral area of the weevil body to be 1 cm^2^, the average attachment force of the weevil measured on the roughest surface (12 µm) in this study is 181 mN, similar to the wind force the weevil is subjected to from the common wind speed of typhoons. This suggests that the weevils probably remain attached to vegetation in the canopy during devastating typhoons, also because the wind pressure might be reduced in forest due to dense vegetation. By contrast, the strongest known adhesion force on smooth surfaces reached by similar adhesion principle among arthropods (89.41 mN, the potato beetle, [33]) is only half of the wind force, assuming the same lateral area. Though it is unclear whether this strong attachment adhesion to smooth surfaces has evolved for the environments on islands or as a co-adaptation for flightless lifestyle, such a strong attachment ability would prevent fatal events, e.g. being blown off into the ocean or puddles, and help to maintain their populations on tropical oceanic islands. Nevertheless, tropical cyclones bring not only violent winds but also torrential rains and the performance of the weevil's attachment system in wet conditions requires further investigation. *Pachyrhynchus* weevils are flightless, but widespread in oceanic islands via passive dispersals potentially through eggs in fruits of floating sea poison trees [15] or eggs in the guts of birds [52]. Though the strong attachment may help the weevils remain on the island during windy events, it may also enhance passive dispersal by attaching to birds (e.g. hummingbird flower mites and swallow bugs; [53,54]), which is potentially the third strategy of cross-island dispersal.

The setae of the teneral weevil have uniform level of sclerotization and a weaker attachment force than that of the mature weevils, yet the teneral weevil still has better attachment ability than most arthropods (electronic supplementary material, information S2). The tarsomeres and setae of the teneral and mature weevils showed similar morphology (figure 2 & S3) and material gradients (figure 3 & electronic supplementary material, figure S4). However, the teneral weevil has relatively less blue colour in the setae (electronic supplementary material, figure S5), indicating proportionally less resilin-like cuticle than mature weevils. This is probably because these soft and resilient materials are presumably deposited in setae after the weevils leave the pupal chambers and are accumulated over post-eclosion maturation. The less deposition of resilin-like materials of both setal bases and tips may decrease the attachment ability of the teneral weevils. However, even though the attachment force is weaker than the mature counterparts by 46% (electronic supplementary material, figure S2), the attachment force of the teneral weevil is still substantially stronger than that of the arthropods by eightfold (on glass surface). This suggests that the flexible bases are responsible for strong adhesion and probably enables the teneral weevils to withstand the severe winds immediately after emergence out of pupal chambers and prevents their softer exoskeleton [17] from physical injuries during a storm.

Even though the clawless weevil has the similar attachment ability as the intact weevils on our tested surfaces (electronic supplementary material, information S1; claw tip radius, 3.02 µm, measured using the same methods as [29]), claws are important for attachment on rough substrates by interlocking the tips with surface asperities [29,55,56]. Plant surfaces are often rougher than the surfaces used in the present study (0.12–4.04 µm). For example, the average roughness of leaf surface of *Vicia faba* is approximately 20 µm [21] and flower petal surface ranges from 5 to greater than 50 µm depending on species [23]. Stem and branch surfaces are presumably rougher, while fruit surfaces could be smoother [57]. The attachment force from adhesive pads may decrease on rough surfaces due to imperfect contact between setal tips and substrate, but can be enhanced by the action of claws. We chose the range of surface roughness that has been commonly used in previous studies for reliable comparisons, though the variations between these surfaces captured mainly the effect of adhesive pads. Nevertheless, future experiments on natural substrates of the weevils could reveal the actual performance they generate in the field.

Much higher attachment ability of the weevil from oceanic islands in comparison with the other arthropods from the mainland could be due to their different habitats or/and behavioural strategies for environmental challenges. For example, flying species could migrate faster and farther, while flat-bodied non-flying species could better hide in crevices. The flightless weevils of oceanic islands remain staying put on abaxial leave side during torrential rains and strong winds in the field (L.-Y.W. personal observation). However, how they respond to extreme conditions, such as strong wind during typhoons, remains unknown. Future investigations on behaviours of the weevils during storms (e.g. what microhabitats and surfaces they stay on) could improve our understanding of behavioural adaptations to extreme conditions. In the current study, we investigated only an insular weevil yet a more thorough comparative study is required to generate a comprehensive understanding of the attachment abilities between island and mainland populations in weevils and insects in general.

## Data Availability

The dataset and R code are available from Figshare: https://figshare.com/s/92639de7480b246e29f5 [58]. Supplementary material is available online [59].
